# Is there a link between motor learning and mirror neuron system: TMS study

**DOI:** 10.3389/fnhum.2025.1650152

**Published:** 2025-09-11

**Authors:** Ekaterina Pomelova, Matteo Feurra, Vadim Nikulin, Alena Grankina, Roman Solodkov, Tamara Banjevich, Evgeny Blagovechtchenski

**Affiliations:** ^1^Centre for Cognition and Decision Making, Institute for Cognitive Neuroscience, HSE University, Moscow, Russia; ^2^Department of Neurology, Max Planck Institute for Human Cognitive and Brain Sciences, Leipzig, Germany

**Keywords:** mirror neurons system, motor learning, transcranial magnetic stimulation, motor resonance, serial reaction time task

## Abstract

**Background:**

The mirror neuron system (MNS) activates during the performance of an action and during the observation of the same action being performed by another. At the motor output level, MNS activation manifests as motor resonance, or a muscle-specific increase in corticospinal excitability during action observation. This study focuses on how and to what extent motor learning alters the initial mirror response and whether the rate of motor learning is associated with pretraining or post-training levels of mirror response. The study involved 23 healthy adults aged 22.7 years on average. The experiment consisted of six sessions. On the first and last days, a transcranial magnetic stimulation session was performed to assess the putative activity of mirror neurons, as reflected in the level of motor-evoked potential facilitation during action observation under various conditions. From the second to the fifth sessions (four sessions), motor learning was performed, as represented in the form of a serial reaction time (SRT) task.

**Results:**

We observed a statistically significant decrease in reaction time during the process of learning within the SRT task and motor facilitation during action observation, thus reflecting putative mirror neuron activity. We found a significant correlation between the learning speed of the non-dominant hand and mirror neuron activation in the dominant hemisphere during the observation of button presses and pinch gestures.

**Conclusion:**

The MNS excitability is not a predictor of motor learning, but motor learning is reflected in the characteristics of the MNS.

## 1 Introduction

In 1992, the first description of mirror neurons in monkeys was published. These neurons are a class of cells located in the premotor area that become active both when the animal performs a goal-directed action (e.g., grasping a piece of food) and when it observes an experimenter or another monkey performing the same or a similar action (di Pellegrino et al., 1992). These neurons were discovered through invasive single-neuron recordings in inferior area 6 (sector F5) (Rizzolatti et al., 1996). In humans, various imaging and electrophysiological modalities have also demonstrated the activation of an analogous brain area during action observation (Kilner et al., 2009; Mukamel et al., 2010; Bonini et al., 2022). Mirror neurons have since been identified in several additional brain regions, leading to the adoption of the broader term “mirror neuron system” (MNS). MNS underlies the phenomenon of motor resonance. Motor resonance is a measurable effect that reflects the activation of the MNS during action observation (Cabinio et al., 2010; Fadiga et al., 1995). Therefore, the term mirror neuron system is appropriate and relevant in this context, as it denotes the neural circuitry responsible for the observed motor facilitation effects. In this study, we hypothesized that changes in MNS activation before and after motor task training reflect the rate of motor learning.

To study the human MNS, researchers use various methods, such as transcranial magnetic stimulation (TMS) and electroencephalography (EEG). When TMS is applied to the primary motor cortex (M1), it elicits motor-evoked potential (MEP), with significantly increased amplitudes during action observation as compared to the rest condition (Fadiga et al., 1995; Alaerts et al., 2009; Strafella and Paus, 2000). In EEG studies, the suppression of the mu rhythm is observed when a person performs, observes, or imagines performing an action (Muthukumaraswamy et al., 2004; Fox et al., 2016).

Although the functions of the MNS are not fully understood, most current evidence suggests that it is involved in the imitation and low-level recognition of object-directed movements, such as grasping a cup (Heyes and Catmur, 2022). Beyond action recognition, several hypotheses propose that the MNS is an evolutionary adaptation for social cognition. It is thought to contribute to understanding others’ intentions, generating empathic responses, and facilitating learning through observation (Rizzolatti and Arbib, 1998; Rizzolatti and Craighero, 2004). Research demonstrates that when a person observes another individual performing an action, a motor resonance effect occurs (Nieto-Doval et al., 2025). Specifically, the observer’s motor cortical areas are activated in a manner similar to when they perform the action themselves (Avenanti et al., 2005; Grèzes and Decety, 2000). For example, studies involving dancers have shown that observing familiar dance movements leads to greater MNS activation compared to unfamiliar ones (Calvo-Merino et al., 2005, 2006). Furthermore, additional research indicates that dancers initially unfamiliar with specific steps exhibit increased activation of the mirror neuron system after undergoing motor training and mastering those steps. This suggests that the MNS is not only involved in action recognition but also plays a role in learning new motor skills through experience (Cross et al., 2006).

This learning mechanism is based on the fact that action observation involves more than just passive visual processing of movement. Instead, it actively engages the observer’s motor system through the process known as motor resonance. During this process, observing an action automatically activates neural patterns corresponding to the execution of similar movements (Gallese et al., 1996; Rizzolatti et al., 1996; Fadiga et al., 2000). In essence, the observer’s brain generates an internal emulation of the motor code associated with the observed action, involving both cortical and subcortical structures. Neurophysiological studies have shown that this process engages cortico-cortical connections between the anterior intraparietal cortex, the ventral premotor cortex, and M1 (Shimazu et al., 2004; Cerri et al., 2003). Importantly, the observation of transitive actions—especially those involving object manipulation—activates motor representations that are specific to the effector used in the action (Fogassi et al., 2001; Buccino et al., 2004). This indicates that the perception of movement triggers not abstract representations of action but detailed motor patterns that include effector specificity, movement direction, and the goal of the action. Consequently, the mirror neuron system provides a neurophysiological basis for learning through action observation by directly linking perception to the motor structures involved in performing the action.

There are several theories of motor learning relating to MNS. One of them is the Associative Sequence Learning (ASL) theory. According to this theory, sensorimotor experience plays a central role. When there is a contingent or predictive relationship between an observed action and an executed action during experience, the connection between them becomes stronger. As a result, associations between the sensory and motor representations of the action are reinforced. This leads to the formation of motor neurons that respond strongly to the sensory stimulus with which they have become associated during the learning process. If the association is made between the observation and the performance of the same action, this motor neuron is now a mirror neuron (Catmur, 2015).

The ASL theory posits that individuals learn by observing their own actions or imitating the actions of others. However, such opportunities are not always available in many real-world learning scenarios. For example, when an individual watches an instructional video about how to throw a ball into a hoop and then practices independently, the visual stimuli differ, but the person may still imagine the same sensory consequences of the movements. This process aligns with the principles of ideomotor theory (Lago-Rodríguez et al., 2014). Most empirical studies have focused on the presentation of egocentric stimuli (i.e., stimuli from the observer’s own perspective), but the extent to which such stimuli accurately reflect learning in real-world contexts remains uncertain, as people often learn through the observation of others, rather than through egocentric viewpoints.

Like the ASL theory, the ideomotor theory assumes that learning promotes the association of sensory and motor codes. However, ideomotor theory states that during learning, additional ideomotor representations are formed that resemble anticipations of the to-be-produced sensory consequences of an action. According to ideomotor theory, these representations primarily serve a motor control function. We control our actions by anticipating their sensory consequences (Brass and Muhle-Karbe, 2014). Moreover, ideomotor theory predicts a specific form of sensorimotor compatibility, namely ideomotor compatibility. A stimulus resembling a sensory action-effect’s anticipation activates the corresponding ideomotor representation. For example, the image of another person opening their hand strongly overlaps with the representation used to control the hand-opening movement. Consequently, ideomotor-compatible stimuli can bypass response selection by directly activating motor programs (Brass et al., 2001).

Our study used ecological stimulus presentation by an actor behind a screen, such as button pressing, pinch-to-grip gestures, and base hand movements, to assess MNS excitability. We then examined the correlation between MNS activation and the speed of motor learning in a serial reaction time (SRT) task. The SRT task was chosen because it is not merely a motor learning task; rather, it can have both motor and perceptual learning components. Recognizing that perceptual learning is a component of the SRT task is important. It explains the shift in brain areas supporting SRT task performance when the perceptual properties of the SRT task are altered (Robertson et al., 2001). Executing the SRT task involves the same muscles required for button pressing, including the flexor digitorum superficialis, flexor digitorum profundus (Wu et al., 2008), and first dorsal interosseus (FDI) (Winges et al., 2013; Carlsen et al., 2008). Performing a pinch gesture involves muscles such as the adductor pollicis brevis, first dorsal interosseous, and flexor pollicis brevis (Lee and Wisser, 2012). Therefore, the observation of button-press actions can be compared with actual button-press performance during the SRT task.

The present study focuses on determining whether there is a relationship between MNS activation, either before or after motor training, and the speed of motor learning. Changes in MNS activity were measured as changes in the amplitude of TMS-induced MEPs in response to the presentation of an ecologically relevant stimulus. The motor task (SRT) required executing the same action observed during the stimulus phase in response to various visual cues. The central hypothesis of our study is that if the MNS is involved in motor learning, then we should be able to predict the rate of motor learning based on MNS activation before training or find a correlation between learning speed and MNS activation following training.

## 2 Materials and methods

### 2.1 Participants

The study included 24 healthy subjects. The number of subjects was chosen based on sample size derived from previous studies (Burgess et al., 2013; Wu et al., 2017; Cavallo et al., 2013; Cross et al., 2009). One participant missed 1 day of the behavioral component of the experiment, and thus, their data were not included in the analysis. Therefore, we have data from 23 participants (16 females, seven males) for analysis. The mean age of the participants was 22.7 years (SD = 2.18). The exclusion criteria included regular sleep of less than 6 h per day, taking stimulants before the experiment, including caffeine, self-reported left-handedness, past history of brain injury or head trauma, being diagnosed with any psychiatric or neurological illness including epilepsy and migraines, family history of epilepsy, taking any prescribed medication, and having metal objects inside the body. All participants read and signed the informed consent form before the experiment. All procedures were approved by the ethics committee of the National Research University-Higher School of Economics (HSE 19/01/2019), Moscow.

### 2.2 Time course of the experiment

**FIGURE 1:**
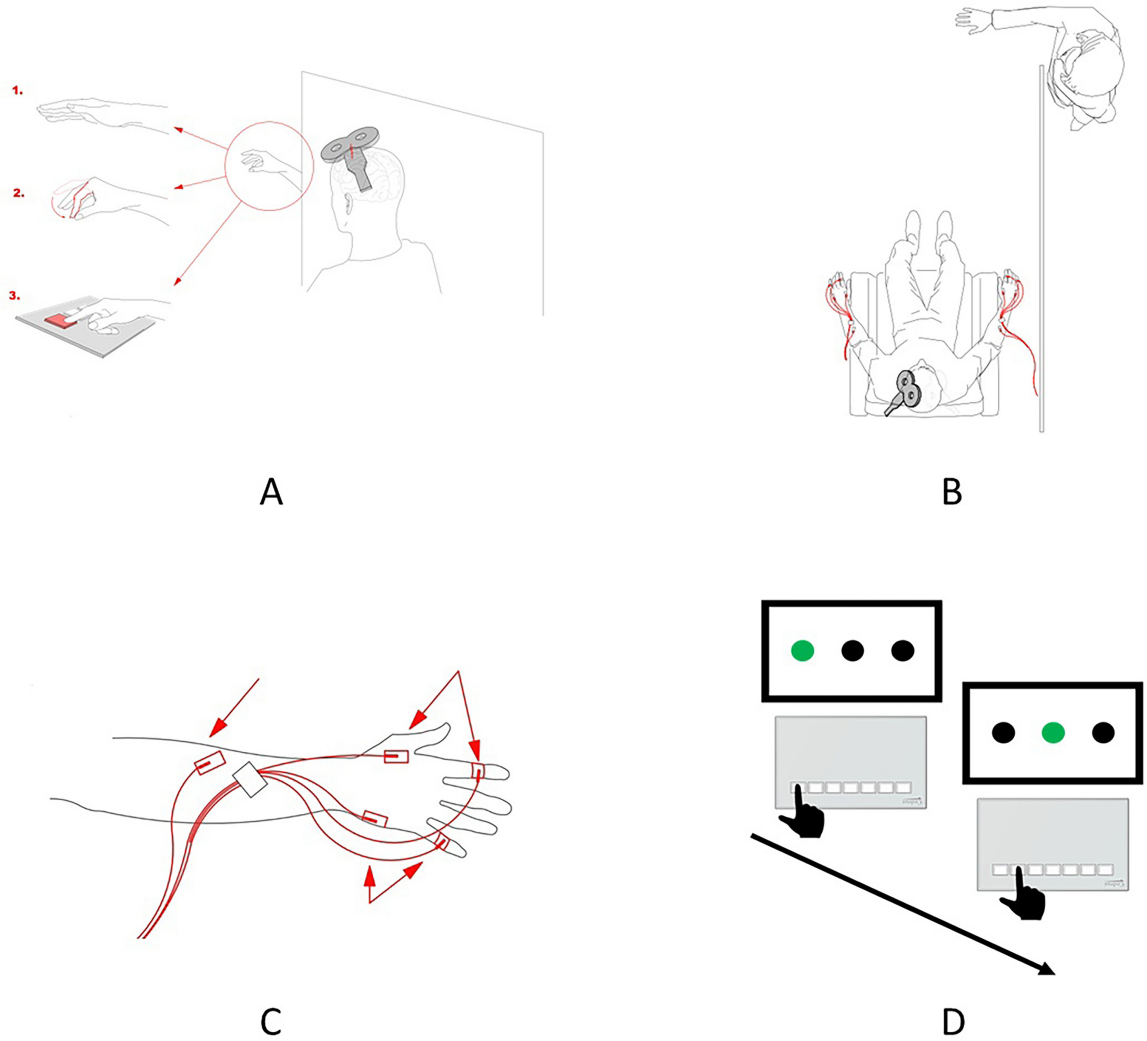
Experimental design. **(A)** Schematic representation of the experimental conditions implemented during the TMS session. Observation of the static hand (1), observation of the pinch-to-grip movement (2), and observation of the button-pressing movement (3). In the preobservation and post-observation baseline conditions, the participants were requested to relax and observe the hand before them. **(B)** Schematic representation of the spatial setup of the experiment. The participant can observe only the hand of the actor, while the remainder of the actor’s body is located behind the curtain and is, thus, invisible to the participant. The red lines depict the location and placement of the EMG electrodes. **(C)** Scheme for the surface electrode placement. Two electrodes were used for recording EMGs in the FDI, two were used for recording EMGs in the ADM, and a ground electrode was placed on the forearm. **(D)** Two sequential trials of the SRT task. The black hand depicts the correct button-pressing movement in the trial, that is, one of the three buttons, which corresponds to the green circle on the screen, should be pressed.

For each participant, the experiment consisted of six sessions. All sessions were held consecutively on separate days. Each participant took part in the experiment for 6 days. As a result, the experiment consisted of 138 sessions. On the first day, a pretraining TMS session was performed to assess the putative activity of mirror neurons, as reflected in the level of motor-evoked potentials (MEPs) facilitation during action observation under various conditions (“Baseline”, “Baseline Hand”, “Pinch”, “Button”, “Baseline Post”; see the description below). The intervals between each TMS condition were randomized between 1 and 5 min to minimize potential carryover effects. These five conditions were implemented during the stimulation of the dominant or non-dominant hemispheres, with the simultaneous EMG recording in the contralateral hand, resulting in ten conditions being performed during the pretraining TMS session. From the second to the fifth sessions (four sessions in total), the motor learning component was performed in the form of an SRT task. The task was implemented separately for both hands (dominant and non-dominant). On the sixth day, a post-training TMS session, which was completely identical to the pretraining TMS session, was held to assess changes in the pattern of motor resonance (Figure 1A). To ensure the participant’s full involvement in observing the action taking place during the TMS session, white screens were placed to limit the participant’s field of vision.

Observing a third party pressing a button partially mirrors the motor task that a participant will perform when learning the SRT task, in which the participant is required to press the response pad buttons corresponding to the color change in a circle displayed on the screen (for more details, see the serial reaction time task section). In a study by Lee et al. (2012), it was demonstrated that observing a third person performing the SRT task via video resulted in a reduction in reaction time and a significant increase in task accuracy, confirming that motor learning can occur through the observation of another individual performing the SRT task.

Additionally, research by Burgess et al. (2013) indicated that observing an action from both an egocentric perspective (i.e., one’s own point of view) and an allocentric perspective (i.e., another person’s point of view) does not lead to a significant difference in the activation levels of human mirror neurons.

### 2.3 TMS session

TMS was applied over the left or right primary motor cortices (M1) through a MagPro X100 (MagVenture) stimulator with a C-B60 Butterfly induction coil (MagVenture). For neuronavigation purposes, the Montreal Neurological Institute template was adjusted to the participants’ individual coordinates with the help of a TMS navigation system (Localite TMS Navigator, Localite GmbH). This approach ensured the consistency of the defined hotspot throughout the experiment and across the sessions. The coil was held tangential to the scalp, with the handle pointing backward and laterally, at a 45° angle from the midline sagittal axis of the participant’s head. Single TMS pulses were delivered to determine the optimal hotspot for the FDI muscle (i.e., the scalp point at which stimulation at the threshold intensity elicited MEPs in the FDI muscle of the contralateral hand). The resting motor threshold (RMT) for the given hotspot was reported as the minimal stimulation intensity that induced MEPs of a minimum of 50 μV in a resting muscle during five out of ten pulses. A stimulus intensity of 120% of the RMT (120% of RMT) was used for experimental purposes. For each condition, 20–30 MEPs were recorded. Before each pulse, the “Go” command was voiced to prepare participants for the focused observation of the action executed. In the case of action observation, pulses were delivered in the middle or final phase of the action. This variability in the timing of the stimulation was introduced to prevent participants from predicting the exact moment of the stimulus application.

The following five conditions were implemented:

(1)    Pre-observation resting state (Baseline), during which the participants were instructed to sit in a relaxed manner, with their eyes open.
(2)    Observation of static hand (Baseline Hand), during which the participants were asked to observe the static hand of the actor (Figure 1A.1).
(3)    Observation of pinch-to-grip movement (Pinch), during which the participants observed the actor who executed the pinch-to-grip movement (Figure 1A.2).
(4)    Observation of button-pressing movement (Button), during which the participants were asked to observe the actors’ hand pressing the button on the keypad (Figure 1A.3).
(5)    Post-observation resting state (Baseline Post), which was the same as the preobservation resting state but was recorded at the end of the experimental session.

All subjects observed the actions during the TMS session from a third-person perspective. Third-person action observation shows poorer results in observation-based learning compared to first-person observation (Yu and Park, 2022). However, our aim was to assess whether baseline MNS activity, measured consistently across participants, could predict motor learning rates rather than to maximize observation-based learning *per se*. Therefore, we do not believe that the subject’s point of view on the presented stimuli is a very critical issue, given that the stimuli were presented in the same way in both TMS sessions.

The order of “Baseline,” “Baseline Hand,” and “Baseline Post” was fixed as presented in the list above to avoid potential long-term excitatory effects on the part of observational conditions on the resting state and control conditions. Two observational conditions, on the other hand, were counterbalanced. There was a rationale behind including these particular conditions. A preobservation baseline was included to assess corticospinal excitability in the resting state. A post-observation baseline recording was added to ensure the absence of significant changes in participants’ corticospinal excitability after the observation-related conditions. Additionally, the observation of the static hand was included to control for potential resting state differences because of the mere observation of a steady hand. Observing the pinch-to-grip and button-pressing movements was expected to induce the mirror-related muscle-specific facilitation of corticospinal excitability, thus allowing to measure motor resonance as an index of mirror neurons’ activity. Both these actions involve the FDI, but not the abductor digiti minimi muscle (ADM); thus, we expected to observe an increase in peak-to-peak MEP amplitude, mainly for the FDI. The difference between these two conditions is that pinch-to-grip movement was observed only during the TMS sessions and was not practiced during the behavioral part of the experiment. In contrast, the button-pressing movement was practiced extensively by participants during the completion of the SRT task.

All five conditions were implemented for both hemispheres and corresponding hands (dominant and non-dominant). The participants observed only the actor’s hand (the rest of the body was behind a curtain), executing actions with the hand corresponding to the one recorded by the participant (Figure 1C). For both sessions (pretraining and post-training), the experimental procedures were the same. To rule out the potential confounding factor of the changed hotspot, the same stimulation spot was used in the pretraining and post-training sessions, and consistency was ensured by saving navigational markers from the first session and using them during the second session.

### 2.4 Electromyography recording

Before applying the surface electrodes, the participants’ skin was cleaned in the desired areas with ethanol (96% solution) and special scrub gel (Nuprep Skin Prep Gel, Weaver, and Company). For EMG recording, surface electrodes (Resting EKG Electrode, 3M Red Dot) were placed on the FDI and ADM muscles, and reference electrodes were placed on the joints of the index and little fingers. The ground electrode was placed proximally on the hand. The FDI was selected as the target because it is directly engaged in the implemented actions, such as pinch-to-grip and button-pressing movements. In contrast, the ADM served as a control since it is not involved in these actions. The EMG was recorded with an ExG AUX box and BrainVision Recorder software.

For the EMG recording, the sampling frequency was set at 5,000 Hz, and the resolution of the signal was set at 0.1 mV. EMG signals were filtered with a hi-pass filter of 10 Hz and a notch filter of 50 Hz (BrainAmp ExG amplifier). Triggers were sent from the TMS stimulator to the BrainVision software to extract the epochs of the EMG recordings in which TMS pulses were applied. The EMG data were processed with the help of MATLAB software and the Berlin Brain–Computer Interface toolbox.

### 2.5 Serial reaction time task

The participants underwent motor training for 4 days in a row. Each day, the SRT task was performed for 10 min (approximately 600 blocks) for each hand (dominant and non-dominant), that is, 20 min per day. The order of task completion regarding the dominant and non-dominant hands was counterbalanced across participants. Each participant had 2 days in which the first hand was dominant and two days in which the first hand was non-dominant. For the SRT task, the Cedrus RB-740 response pad was used. The task was as follows: The participants were seated in front of the computer screen. On the screen, three black circles located horizontally were presented. When one of the circles was lit with green light, the participant was instructed to press one of three buttons on the keypad corresponding to the green circle (Figure 1D). If the participant pressed the wrong button, the next trial was presented. The hand of the participant and the keypad were positioned in such a way that pressing the button would require the involvement of the FDI muscle, but not the movement of the entire palm. The reaction time (RT) was recorded as the main dependent variable with which to assess the learning pattern. The code for the SRT task was implemented with the Presentation software program.

### 2.6 Data analysis

#### 2.6.1 Learning estimation

Behavioral data from the Serial Reaction Time (SRT) task were analyzed to verify whether the experimental manipulation effectively induced motor learning. The primary dependent variable was reaction time (RT) for button presses. The independent variables were day (1, 2, 3, or 4) and hand (dominant or non-dominant). To assess changes in RTs across the four training days and examine potential intermanual asymmetries, a two-way repeated-measures ANOVA was conducted: 4 (days) × 2 (hand).

The sphericity assumption was tested using Mauchly’s test. When violations were detected, degrees of freedom were corrected using Huynh–Feldt estimates of sphericity. In addition to RTs, accuracy was assessed for a subset of participants (*n* = 11) by calculating the ratio of correct responses to total responses.

A learning rate index was computed for each hand as the ratio of mean RT on Day 1 to mean RT on Day 4. Higher values indicated greater learning.

#### 2.6.2 MEP extraction and estimation

As for the EMG data, the peak-to-peak amplitude of the MEPs was used as an index of corticospinal excitability and the main dependent variable in this part of the study. During preprocessing, which was carried out using the Berlin Brain–Computer Interface toolbox (Blankertz et al., 2016) in MATLAB, high-pass (15 Hz) and notch (50 Hz) Butterworth filters were applied. Epochs containing TMS-induced MEPs were then extracted. Finally, the peak-to-peak amplitudes of the MEPs were measured in both recorded muscles. Next, a statistical analysis of the acquired data was performed. At this stage, our independent variables were as follows: condition (“Baseline”, “Baseline Hand”, “Pinch”, “Button” or “Baseline Post”), session (pretraining or post-training), and hemisphere (dominant or non-dominant). MEPs in the “Baseline” and “Baseline Post” conditions were compared to rule out potential changes in general corticospinal excitability during the TMS session. To accomplish this, we implemented a three-way repeated-measures ANOVA: 2 (condition: “Baseline” or “Baseline Post”) × 2 (session: pretraining or post-training) × 2 (hemisphere: dominant or non-dominant). Corticospinal excitability facilitation, which was used as an index of motor resonance, was calculated as the percentage ratio of the MEP amplitude in each condition to the MEP amplitude in the baseline condition, which was collapsed, or not, with the “Baseline Post” condition depending on the outcome of the baseline comparison (see below). Then, after normalization, the ratio of the MEP amplitude in each condition was used as the main dependent variable. To assess the differences in the change in corticospinal excitability, a three-way repeated measures ANOVA was performed on the data acquired from the FDI muscle: 3 (condition: “Baseline Hand”, “Pinch” or “Button”) × 2 (session: pretraining or post-training) × 2 (hemisphere: dominant or non-dominant). Since each factor had only two levels, no sphericity corrections were necessary. Based on the non-significant results of this analysis, the Baseline and Baseline Post conditions were collapsed into a single baseline measure for each muscle. To investigate the muscle specificity of the observed effects, the same analysis was performed on the data obtained from the ADM muscle. For the subsequent *post hoc* analysis, a paired-samples *t*-test with Holm–Bonferroni correction was implemented.

### 2.7 Statistics

The associations between the motor learning rate and MEP facilitation in the pretraining and post-training sessions were assessed with Spearman’s correlation. The learning rate was normalized as the ratio of mean RT on the first day to mean RT on the fourth day for each hand. This ratio was chosen for comprehensibility: The higher the ratio, the more pronounced the learning is. Comparisons were made between the levels of motor facilitation (for both sessions in both hands during observation of pinch-to-grip and button-pressing movement—eight conditions) and learning rates (in dominant and non-dominant hands—two conditions), resulting in 16 comparisons in total. The multiple comparisons issue was accounted for with the help of a Bonferroni correction. All statistical analysis, data handling, and visualization were performed in RStudio software. For the statistical analysis itself, the ez, corrplot, and multcomp packages were used. Data handling was mainly performed with the data.table package. Finally, the ggplot2 package was used for data visualization.

A correlation analysis was performed to examine the relationship between the activation of MNS when the subject was observed pressing a button and pinching with the dominant and non-dominant hands. The analysis was conducted using Microsoft Excel, employing the CORREL function to calculate the Pearson correlation coefficient (r).

## 3 Results

### 3.1 Learning estimation

**FIGURE 2:**
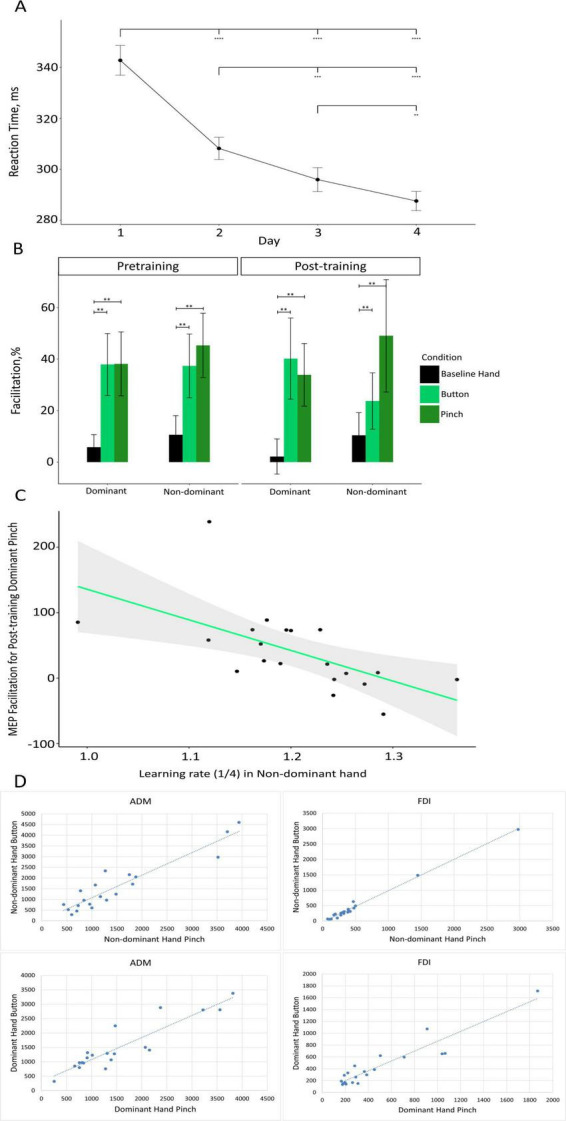
**(A)** Results of the SRT task. Mean RT (in ms) is presented for each of the 4 days of the training. Black circles depict the mean RT for each day. Error bars depict the standard error of the mean (**p < 0.01, ***p < 0.001, ****p < 0.0001). **(B)** Changes in MEP amplitude as compared with baseline, in percentage terms, during the observation of the static hand (“Baseline Hand” – black), the observation of the button-pressing movement (“Button” – light green), and observation of the pinch-to-grip movement (“Pinch” – dark green) in the dominant and non-dominant hemispheres (on the x-axis) for the pretraining (left) and post-training (right) sessions. Data are presented as means ± standard errors of mean (**p < 0.01). **(C)** Scatter plot depicting the association between the learning rate in the non-dominant hand (on the *x*-axis) and MEP facilitation during the observation of pinch-to-grip movement in the dominant hand during the post-training session (on the *y*-axis). The green line represents the fitted linear function of the data, and the gray area represents the 95% confidence level interval. **(D)** Scatter plot showing the relationship between the activation of the MNS when a subject observes pressing a button and pinching for the dominant and non-dominant hemispheres during the pretraining TMS session.

Regarding the analysis of the SRT task, Mauchly’s test indicated that the assumption of sphericity was violated for the day variable (*W* = 0.48, *p* = 0.009) and the interaction between the day and hand variables (*W* = 0.19, *p* < 0.0001). Thus, degrees of freedom were corrected using the Huynh–Feldt estimates of sphericity for day (*e* = 0.74) and the interaction of day and hand (*e* = 0.55). A two-way ANOVA revealed significant main effects on the part of day (F (2.22, 48.80) = 91.59, *p* < 0.0001, h^2^ = 0.309) and hand (F (1.22) = 5.62, *p* = 0.027, h^2^ = 0.011), while the interaction effect of these two variables (F (1.65, 36.21) = 0.74, *p* = 0.530, h^2^ = 0.002) was not significant. Based on this pattern of effects, performance was different each day, with a gradual decrease in RTs being observed across the sessions (Figure 2A), and this suggests that our experimental manipulation was successful in terms of inducing a pronounced motor learning effect in participants. Moreover, the significant main effect of hand indicates that performance on the SRT task was generally better with the dominant hand than with the non-dominant one. However, the absence of an interaction effect indicates that the learning rate, which is characterized by a gradual decrease in RTs across experimental sessions, did not differ between the dominant and non-dominant hands. For the subset of participants in whom accuracy was recorded (*n* = 11), mean accuracy was 95.4% (SD = 1.1%), indicating a high level of task performance.

### 3.2 MEP estimation

#### 3.2.1 Comparison of baselines

The three-way repeated-measures ANOVA revealed no significant main or interaction effects for baseline MEP amplitudes in either the FDI or ADM muscles. Specifically, the condition effect (Baseline vs. Baseline Post) was not significant for FDI (F(1, 21) = 0.02, *p* = 0.878, h^2^ = 0.00001) or ADM (F(1, 21) = 1.65, *p* = 0.212, h^2^ = 0.001). Thus, the baseline values were collapsed for subsequent analyses.

#### 3.2.2 Mirror effect

Post hoc comparisons indicated that corticospinal facilitation during static hand observation (*M* = 7.23%, SD = 33.78%) was significantly lower than during both button-pressing observation (*M* = 34.80%, SD = 61.10%; t(22) = 3.38, *p* < 0.01) and pinch-to-grip observation (*M* = 41.60%, SD = 72.23%; t(22) = 3.31, *p* < 0.01). The difference in the facilitation of excitability during the observation of the button-pressing movement and pinch-to-grip movement was not significant (t(22) = 1.00, *p* = 0.33). Facilitation data for the conditions only (merged across sessions and hemispheres) are depicted in Supplementary Figure 1.

#### 3.2.3 Differences in the mirror effect between sessions

A three-way repeated-measures ANOVA on corticospinal excitability in the FDI muscle revealed a significant main effect of condition (F(2, 44) = 8.98, *p* < 0.001, h^2^ = 0.062). No significant effects of session or interactions were found. A one-way repeated-measures ANOVA on data collapsed across sessions and hemispheres confirmed this effect (F(2, 44) = 8.98, *p* < 0.001, h^2^ = 0.112). The visualization of MEP facilitation across conditions, sessions, and hemispheres is presented in Figure 2B.

#### 3.2.4 Changes in MEP amplitudes in the ADM muscle

The following analysis assessed changes in the corticospinal excitability of the ADM muscle. A three-way repeated measures ANOVA showed the absence of any significant effects, main or interaction alike. Most importantly, the main effect on the part of condition was not significant (F(2, 44) = 1.29, *p* = 0.285, h^2^ = 0.0066), demonstrating that the facilitation of MEP amplitude does not change across conditions for the ADM muscle. Based on this, any effect we observe only in the FDI muscle (target muscle) most likely reflects the activity of the MNS. No changes in the ADM muscle (control muscle) were found, supporting the specificity of potential effects.

### 3.3 Analysis of the correlation between MEPs and learning

We attempted to investigate the relationship between the speed of motor learning and the levels of motor resonance in pretraining and post-training sessions (Figure 2C). Significant correlations were found between the rate of non-dominant hand learning and the activation of mirror neurons in the dominant hemisphere after training in button pressing *p* = 0.03 and pinching *p* = 0.003.

A correlation analysis was used to investigate the relationship between activation of the MNS when the subjects observed pressing a button and MNS activation when the subjects pinched with their dominant or non-dominant hands. The Pearson correlation coefficient (r) was greater than 0.9 for each pair of correlations, which indicates a strong positive linear relationship (Figure 2D).

## 4 Discussion

The present study explored motor learning and its relationship with motor resonance and mirror neuron activity. The SRT task was employed over 4 days, leading to a significant decrease in RT, indicating successful motor learning. The observation of button-pressing and pinch-to-grip movements induced mirror neuron activity, as evidenced by MEP facilitation. We also found a significant correlation between the learning speed of the non-dominant hand and mirror neuron activation in the dominant hemisphere during the observation of button presses and pinch gestures.

We would like to highlight one feature of this work: Because of the explorative character of our study, it included many statistical comparisons between training days and MNS activation for the dominant and non-dominant hemispheres under three conditions. Therefore we adjusted for multiple comparisons, considering all comparisons in order to avoid type I error. If several conditions were dropped, the number of significant effects would increase. However, we considered it necessary to preserve the entire picture and present all the data.

### 4.1 Motor learning

An analysis of SRT task results revealed a significant decrease in the mean RT across the four training days, indicating that the implemented design was sufficient in terms of inducing motor learning in the participants. We see a decrease in reaction time during training already on the 2nd day, an accepted behavioral indicator of motor learning. This observation aligns with previous studies where it was shown that the mastery of the SRT occurs even after its first completion (Kwon et al., 2015), while other experiments used 3 days of SRT training (Olivier et al., 2021), there are also studies showing that 4 days of practice is really enough to master the skill of matching a keystroke and a visual cue (Vleugels et al., 2020). Based on this prior evidence and the observed behavioral improvements in our own dataset, we considered a 4-day training period to be appropriate and sufficient for inducing measurable learning effects in the SRT task.

Even though the main effect on the part of hand on RT was also significant, the absence of a significant interaction effect signifies that the learning rate itself was not different between the two hands. In sum, these data suggest that learning did occur as a result of the completion of the implemented SRT task, putatively inducing a sensorimotor association between button-pressing movement and simple geometric stimuli (green circles on the screen), and that the rates of this type of learning were not different between the dominant and non-dominant hands. It is possible that performance on the SRT task was generally better for the dominant hand because of control asymmetries, which can affect adaptive processes. It has previously been shown that there is no difference in the speed or final degree of adaptation between hands, suggesting that visuomotor adaptation may occur similarly for both hands (Mutha et al., 2012).

### 4.2 Mirror effect

Post hoc comparisons implemented for the electromyography data from FDI muscle collapsed across hands and sessions demonstrated that MEP facilitation was significantly different for observing button-pressing and pinch-to-grip movements as compared with the observation of a static hand. This effect suggests that the implemented setup was adequate in terms of eliciting motor resonance (mirror effect), which manifested as a muscle-specific increase in corticospinal excitability during the observation of the movement being performed by another individual. The muscle specificity of this effect was demonstrated by the absence of any significant differences across conditions for MEP facilitation in the ADM muscle. Mirror neurons are activated when an action is observed in areas of the cerebral cortex responsible for the muscles involved in the viewed activity (Heyes and Catmur, 2022; Wurm and Caramazza, 2019; Wurm and Lingnau, 2015). Thus, our results agree with a large body of literature on this topic. Thus, our results are consistent with a large body of literature on this topic. For example, a study by Fadiga et al. (1995) showed that the amplitude of the MEP increases during action observation compared to the observation of static objects (Fadiga et al., 1995). But Stefan et al. (2005) showed that observing movements leads to the formation of a stable specific memory trace in the form of representations of movement that resemble those formed during physical practice. Moreover, physical training alone leads to an increase in MEP amplitude when recorded without concurrent action observation (Stefan et al., 2005). Therefore, these data are inconsistent with ours, since we did not find significant changes in the amplitude of the MEP before and after a motor learning session.

We also found a significant correlation in the pretraining session between MNS activation while observing button presses and pinch gestures for both the dominant and non-dominant hands in the APB and FDI muscles. This indicates a generalized effect on the part of MNS activation across tasks. Based on this result, we conclude that mirror neurons are not task specific in their activation, suggesting that a task need not visually replicate the observed action. Therefore, we emphasize that in this study, using the SRT task is appropriate and well justified.

### 4.3 Correlation between motor learning and MEP facilitation

We hypothesized that motor learning during the SRT task would be correlated with the facilitation of corticospinal excitability during action observation in the pretraining session, reflecting a connection between the motor resonance phenomenon and motor learning processes. However, all corresponding correlations were insignificant, not only for the pretraining session but also for the post-training session, thus demonstrating the absence of an association between motor learning rate and changes in corticospinal excitability during action observation in this experimental setup. This is inconsistent with some of the previous studies. Studies have shown that changes in the excitability of the corticospinal system are the result of learning to move (Koeneke et al., 2006; Cirillo et al., 2011; Bagce et al., 2012). Variations in experimental design may account for the inconsistencies between our findings and those reported in prior studies. One distinctive feature of our study is the use of motor learning based on the same movement that was presented during the assessment of MNS activation, while the visual stimuli used during the learning phase were different. Most studies have focused on learning through observing egocentric stimuli, which represents a significant difference from our approach. In previous studies, motor learning was based on nonmatching action and observation stimuli (learning to perform index-finger abduction in response to the observation of little-finger abduction), while in our experiment, training was based on novel simple geometric stimuli (green circles on the screen). Thus, the training was purely additive nature and did not cause a mismatch between the observed and performed actions. In our study, motor learning was performed over 4 days, while in most other studies, it was performed over 2 days (Cavallo et al., 2013; Conty et al., 2012; Catmur et al., 2011), which represents a significant difference in experimental design and may have affected the results of the study.

In our study, we used different visual stimuli to assess MNS activation and motor learning; however, the observed actions engaged the same muscles as those involved in the motor task. Additionally, performing the SRT task requires the involvement of brain regions such as the prefrontal cortex; motor cortical areas; and subcortical structures, including the striatum and cerebellum (Robertson et al., 2001; Torriero et al., 2004; Pascual-Leone et al., 1993). These same brain areas are activated during the observation of button presses with the right index finger, including the left primary motor cortex, supplementary motor area, ventral premotor cortex, basal ganglia, bilateral anterior cerebellum, claustra, dorsal premotor cortex, dorsolateral prefrontal cortex, right inferior parietal lobule, insula, and inferior frontal gyrus (Turesky et al., 2018). This convergence of findings supports the methodological validity of our experimental design.

Our data have demonstrated a correlation between the learning speed of the non-dominant hand and the activation of the MNS of the dominant hemisphere after the motor learning. The MNS might play a role in facilitating the transfer of learned motor skills or motor representations between hemispheres. Since the MNS is involved in action observation and internal simulation, enhanced MNS activity in the dominant hemisphere could reflect a mechanism of motor prediction or reinforcement that aids in consolidating newly acquired skills of the non-dominant hand. This is consistent with models of bilateral motor control, where the dominant hemisphere supports learning through predictive coding and simulation. The model proposed by Mutha et al. (2012) suggests that the left hemisphere provides predictive control mechanisms that determine certain aspects, such as the direction of movement for both the contralateral and ipsilateral arms. At the same time, the right hemisphere also contributes to positional control mechanisms during the movements of either arm (Mutha et al., 2012). This separation of functions may be a way to ensure that none of the processes are completely compromised—the dominant hemisphere can ensure the precise execution of a task. In contrast, the nondominant hemisphere gradually learns to improve movement planning. Thus, the correlation between nondominant hand learning and dominant hemisphere mirroring neuron activation may result from the nondominant hemisphere learning to improve movement planning (Mutha et al., 2012). Another potential role of the MNS could be in error monitoring during learning. After training, increased MNS activation might reflect a heightened sensitivity to action-related sensory feedback, allowing for more precise motor planning adjustments, particularly for the non-dominant hand, which has lower baseline proficiency. Also, the presence of this ipsilateral interaction observed through the correlation between nondominant hand learning and mirror neuron activity in the dominant hemisphere after learning may be related to the ipsilateral component of the corticospinal system. The function of this is currently not known.

Neuroimaging studies have revealed activity in the precentral gyrus ipsilateral to the side of hand movement, especially during complex finger movements, in healthy adults and after stroke recovery (Cramer et al., 1999; Weiller et al., 1992). In addition, TMS can cause hand movements ipsilateral to the stimulated hemisphere (Wassermann et al., 1994). The precentral gyrus areas on the ipsilateral side of the arm movement were interpreted as part of the primary motor areas (Brodmann area 4). This interpretation was based on studies conducted in nonhuman primates that clearly demonstrate ipsilateral or bilateral motor representations within the primary motor areas (Gentilucci et al., 1989). The ipsilateral interaction shown in the present study is consistent with the results of previous functional magnetic resonance imaging studies in the ventral premotor areas involved in distal finger movements (Ehrsson et al., 2000). These studies show that accurate grip performance is associated with stronger signaling in the inferior frontal gyrus, ipsilateral to the responsive hand, and that the ventral premotor cortex contains ipsilateral representations of the fingers (Cramer et al., 1999; Hanakawa et al., 2005).

It is also possible that our results are related to the fact that the nondominant hand is more sensitive to learning than the dominant hand. When learning new motor skills with the nondominant hand, a greater number of brain regions are activated as compared to during training the dominant hand (Kirby et al., 2019). Also, in right-handed subjects, left hemisphere function is associated with both right- and left-hand movements, suggesting that this hemisphere has bilateral involvement in motor control (Grafton et al., 2002). Thus, this study shows that the MNS activation is not able to predict the rate of motor learning but that there is a correlation between the learning speed of the non-dominant hand and the activation of the MNS of the dominant hemisphere after motor learning.

The limitations of our study, such as the specific experimental design chosen and the focus on a novel set of stimuli, highlight the need for further investigations to elucidate the complex relationship between motor learning, mirror neuron activity, and the modulation of motor resonance. Future studies employing diverse methodologies, larger sample sizes, and different learning paradigms are warranted to expand our understanding of these interconnected phenomena. Also, our study was conducted in healthy young participants, and future studies may explore similar mechanisms in older adults.

## 5 Conclusion

In the current study, we investigated whether the rate of motor learning could be predicted based on MNS activation prior to training, as well as whether a correlation could be found between learning speed and MNS activation following training. We observed a statistically significant decrease in RTs during the learning process in the SRT task, as well as differences in motor facilitation during action observation as compared with observing a static hand. This reflects the putative activity of mirror neurons. However, our data demonstrated a correlation between learning speed for the nondominant hand and MNS activation in the dominant hemisphere following motor learning. These findings have broad implications for our understanding of the complexities of motor learning and the intricate interplay between the mirror neuron system and motor processes in the brain.

## Data Availability

The raw data supporting the conclusions of this article will be made available by the authors, without undue reservation.
