# Supplementary material for: Is there a link between motor learning and mirror neuron system: TMS study
Source: Front Hum Neurosci. 2025 Sep 11;19:1650152. doi: 10.3389/fnhum.2025.1650152 (PMC12460307; doi:10.3389/fnhum.2025.1650152)
Supplement: Supplementary file 1 [file Table_1.docx]

Supplementary Material

**
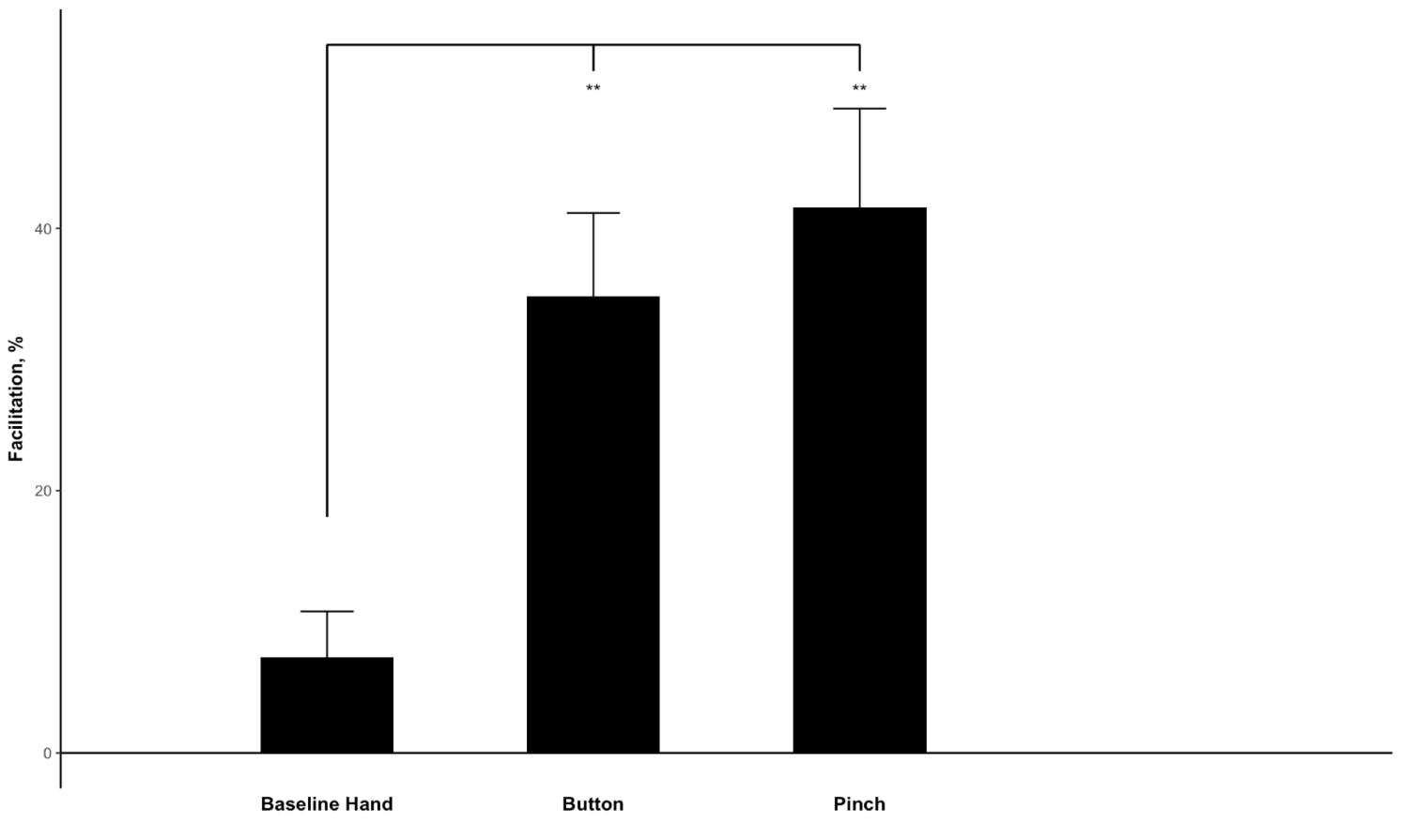
**

**Supplementary Figure 1.** Changes in MEP amplitude in the nondominant hemispheres compared with baseline (in %) during the observation of the static hand (Baseline Hand), observation of the button-pressing movement (Button), and observation of the pinch-to-grip movement (Pinch). Data are presented as mean ± standard error of mean (** – p < .01)
